# Experimental Investigation on the Influence of Crack Width of Asphalt Concrete on the Repair Effect of Microbially Induced Calcite Precipitation

**DOI:** 10.3390/ma16093576

**Published:** 2023-05-07

**Authors:** Ling Fan, Jinghong Zheng, Shuquan Peng, Zhize Xun, Guoliang Chen

**Affiliations:** School of Resources and Safety Engineering, Central South University, Changsha 410083, China; pqrfanlinger@csu.edu.cn (L.F.); jinghong.zheng@csu.edu.cn (J.Z.); xunzhize@csu.edu.cn (Z.X.); chenguoliang0216@163.com (G.C.)

**Keywords:** microbially induced calcite precipitation, asphalt concrete, cracks, uniaxial compressive strength, fibers, spherical calcite

## Abstract

The appearance of cracks is one of the reasons that affect the performance of asphalt pavement, and traditional repair methods have the potential problem of causing adverse effects on the environment. In this paper, an environmentally friendly method for asphalt concrete crack repair was investigated using microbially induced calcite precipitation (MICP) for asphalt concrete cracks of different widths (0.5 mm, 1.0 mm, 1.5 mm, and 3 mm), and the effectiveness of repair was evaluated using nondestructive and destructive experiments. A varied ultrasonic pulse velocity was used to evaluate the healing process, and it was found that the samples with an initial crack width of 0.5 mm showed the most significant increase in wave velocity of 18.06% after repair. The results also showed that the uniaxial compressive strength and indirect tensile strength of the MICP-repaired samples recovered up to 47.02% and 34.68%. Static creep test results showed that MICP-repaired samples with smaller width cracks had greater resistance to permanent deformation. The results of uniaxial compressive strength tests on larger width (3 mm) cracks repaired by MICP combined with fibers showed that the strength of the samples was significantly increased by the addition of fibers. In addition, the SEM/EDS results showed that the MICP products were spherical calcite particles with a particle size distribution from 0 to 10 μm. This study shows that MICP has some potential for repairing cracks in asphalt concrete of different widths within the range investigated.

## 1. Introduction

Asphalt pavements are widely used in traffic engineering all over the world because of the advantages of a short construction period, a smooth surface, and comfortable driving [1]. There are two main types of asphalt pavement cracks: load and non-load cracks. Cracks are almost inevitable in asphalt pavement structures, and long-term traffic loading is an important cause of cracks [2]. Studies have shown that pavement deterioration and cracking can lead to a reduced engineering application performance of asphalt pavements, such as indirect tensile strength [3] and permanent deformation [4].

There are generally two types of solutions for this pathology. One acts before the development of cracks and the other after the appearance of cracks, the former being a proactive way to reduce the possibility of cracks and the latter being a reactive repair after the cracks appear. The first method is to enhance the crack resistance of the asphalt mix, with the effect of preventing the extension of cracks or reducing the likelihood of cracks appearing. This approach includes enhancing the bonding effect of the binder (with the addition of asphalt mastic, rubber, etc. [5,6,7]) and enhancing the properties of the asphalt concrete aggregate (with the addition of polyester, lignin, glass, basalt fibers, etc. [8,9,10,11,12]). When cracks appear in asphalt concrete, the traditional methods of treating cracks are heat-induced self-healing, crack sealing, or filling with organic asphalt-based materials [13,14]. Crack sealers are hardly cost-effective because of their poor treatment performance, high maintenance costs, and high environmental risks [15,16].

Recently, promising results have been achieved in eco-friendly fracture repair using biocolloid technology. Inorganic materials precipitated by microbially induced calcite precipitation (MICP) for the effective repair of cracks in cement concrete, brick, mortar, and rock have been extensively investigated due to their environmental friendliness and low-carbon nature [17,18,19]. The basic principle is to drop a biological agent into an already existing crack in the substrate material. After the biological agent enters the crack, the bacteria contained in the biological agent solution induce the formation of calcium carbonate precipitation by metabolizing the calcium ions and urea in the surrounding environment (cementing solution) [20]. However, the application of MICP in asphalt concrete crack filling has rarely been reported so far.

It has been shown that pure inorganic materials can be used to rapidly repair asphalt concrete cracks in municipal pavement manhole structures [16]. In addition, there have been attempts to combine MICP and asphalt mixtures. The application of microbial technology in asphalt mixtures was first used for modified cold asphalt emulsion mixes (CAEMs) [21]. By comparing the MICP method for CAEMs through two scenarios, it was found that the MICP method could significantly improve the mechanical properties of CAEMs. Among the studies on the moisture sensitivity of CAEMs, it was also found that the resistance to moisture damage was significantly improved after treatment with the MICP technique [22]. The above studies showed that MICP products have some compatibility with asphalt mixtures. Theoretically, it is possible to repair cracks in asphalt concrete using the MICP method.

In this paper, the effect of MICP on the repair effect of cracked asphalt concrete of different widths was investigated experimentally, and the repair performance of cracked asphalt concrete, including uniaxial compressive strength, indirect tensile strength, and permanent deformation, was evaluated. In addition, the physical morphology and chemical components were analyzed using scanning electron microscopy (SEM) and energy dispersive spectrometer (EDS) analysis.

## 2. Material and Sample Preparation

### 2.1. Sample Preparation

The binder used for the asphalt concrete samples was cationic emulsified asphalt (52 mm needle penetration, 66 cm ductility, and 50.5 °C softening point) produced by Dongguan Dongjiao Asphalt Co. The aggregates for the asphalt concrete samples were crushed gravel, mineral powder, and coarse and medium-fine sand, with 10% asphalt content for each sample. Based on the Talbot classification method [23], the mass ratio of five sizes of particles *P_i_* (*i* = 1, …, 5) is as shown in Equation (1):

(1) $$P_{i}=\frac{M_{i}}{M}={\left(\frac{d_{i}}{d_{\max}}\right)}^{n}$$

where *d*_max_ is the maximum size of the particle and *n* is the Talbot index (*n* < 1).

The total mass of each sample was 800 g. The grading of the aggregates at a Talbot index of 0.5 is shown in Table 1.

To investigate the effect of pre-crack width on MICP repair, intact samples and samples with crack widths of 0.5 mm, 1.0 mm, and 1.5 mm were designed for direct MICP repair, as well as samples with crack widths of 3 mm for MICP repair with fibers. It has been shown that the maximum crack depth of asphalt concrete in the field is less than 50 mm [24]. In this paper, for small-width (≤1.5 mm) cracks, the crack depth was set to 30 mm. For cracks of large width (3 mm), the crack depth was set to 40 mm. The length of the precast cracks used was fixed at 40 mm.

NCHRP Report No. 425 [25] was used to prepare the sample. First, the aggregates were weighed with an electronic scale (according to Table 1) and stirred clockwise and counterclockwise for 30 s, and then emulsified asphalt was added to the aggregates and stirred with the same mixing method. The mixture was then poured into a steel mold with 70.7 mm sides and the samples were compacted with a hammer. Next, a PVC hard plastic sheet was embedded in the top center of each sample and the PVC sheet was pulled out with tweezers after 1 h. After 48 h, the samples were de-molded and dried in a programmable temperature and humidity tester (TH-80CH) at 60 °C for 24 h. In this study, 15 samples of each crack width (0.5 mm, 1.0 mm, 1.5 mm, and 3 mm) were prepared, for a total of 60 samples with cracks. In addition, 12 complete samples without cracks were used as control.

### 2.2. Bacterial Culture and Cementing Solution

The bacteria used in this experiment were Bacillus pasteurii because of its significant advantages, such as easy extraction and nonbiological pathogenicity. The composition of the liquid nutrient medium used in the experiment was urea (20 g/L), soy protein (5 g/L), casein (15 g/L), and sodium chloride (5 g/L), and the pH of the medium was adjusted to 7.3 with NaOH. After the nutrient medium was prepared, the bacteria were inoculated into the culture medium at a rate of 1%. Finally, the inoculated bacterial culture was put into a constant temperature shaker and incubated at 30 °C and 130 rpm for 24 h. The OD600 and conductivity were measured every eight hours until their OD600 and conductivity reached about 2 and 20, respectively. An equimolar urea–calcium chloride solution with a concentration of 1 M was used as the MICP cementing solution.

### 2.3. Crack Repair Methods

The repair method varies depending on the width of the crack.

**Figure 1:**
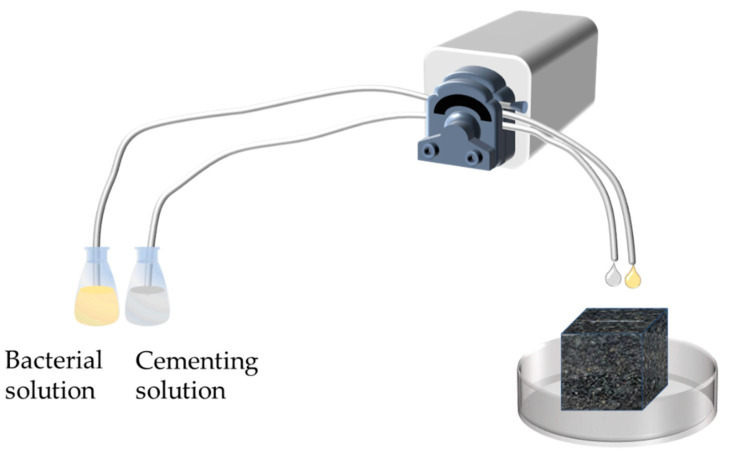
Schematic diagram of MICP grouting.

For small-width cracks (≤1.5 mm), precast cracked asphalt concrete samples were repaired by peristaltic pump drip injection. A BT100M peristaltic pump was used to simultaneously pump 10 mL of bacterial solution and cementing solution into the cracks at a rate of 3.0 rpm for eight days. The crack repair is shown schematically in Figure 1.

**Figure 2:**
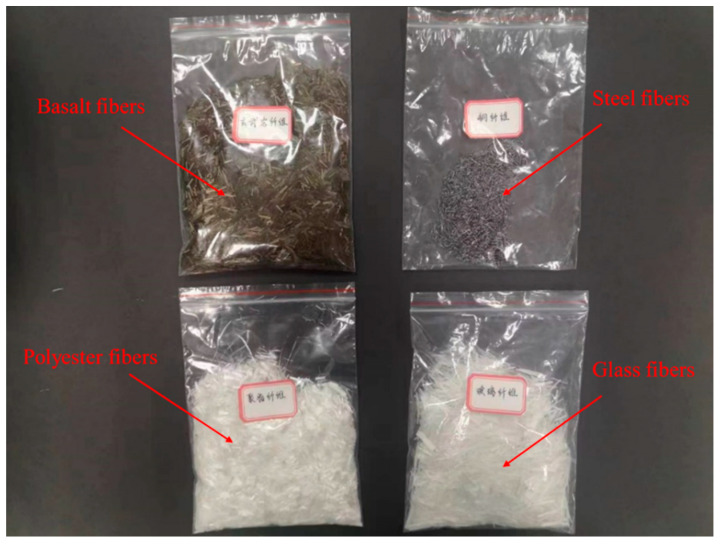
Fibers.

For samples with large-width cracks (3 mm), the MICP repair process used a peristaltic pump to simultaneously pump 20 mL of bacterial solution and 20 mL of the cementing solution into the cracks at 20.0 rpm, and the fibers (basalt fibers, polyester fibers, glass fibers, and steel fibers), as shown in Figure 2, were added separately to the cracks, along with the slurry solution, while grouting. In order to ensure the fibers were evenly distributed in the cracks, the fibers were slowly moved in place using a fine iron rod.

## 3. Evaluation of Repair Effect

Table 2 shows the experimental procedures of this study.

### 3.1. Compressive Strength

**Figure 3:**
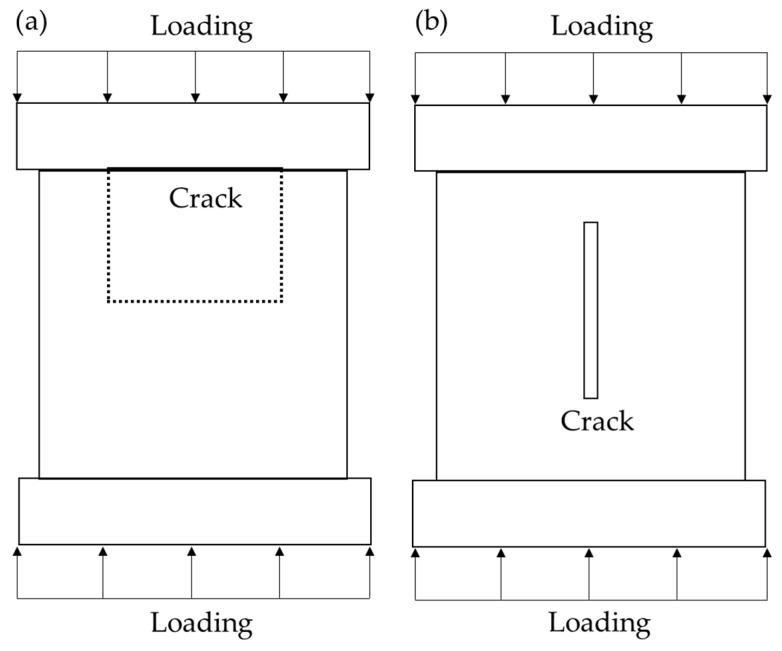
Schematic of loading method: (**a**) small-width crack (≤1.5 mm); and (**b**) large-width crack (3 mm).

**Figure 4:**
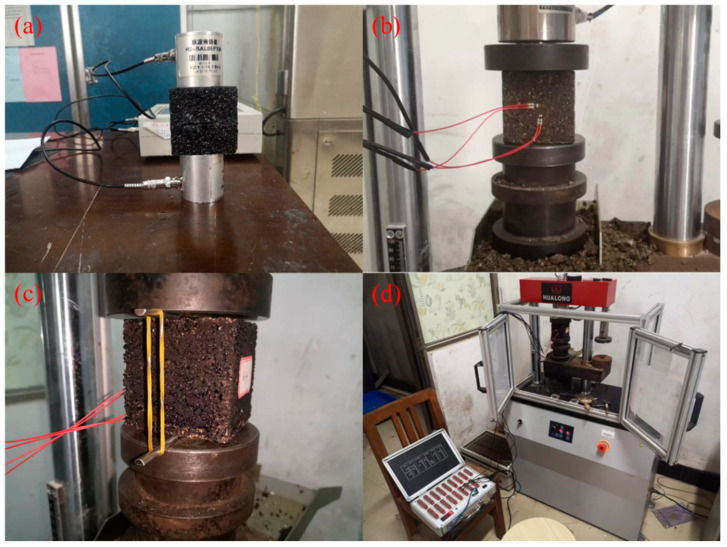
Test procedures: (**a**) ultrasonic pulse velocity; (**b**) UCS test; (**c**) ITS test; and (**d**) static creep test.

The effect of MICP repair on the strength of small-width asphalt concrete cracked samples was determined using indirect and direct methods. For the indirect method, a conventional nondestructive test method called ultrasonic pulse velocity (UPV) was used to assess the degree of internal damage [26]. Prior to testing, petroleum jelly was applied to two opposing planes of the sample parallel to the fracture. A pair of transmitter and receiver transducers of the rock acoustic parameter tester (HS-YS4A) were placed close to these two planes for transmitting and receiving ultrasonic pulses. The ultrasonic velocity is the length of the path between the two transducers divided by the time for the ultrasonic pulse to pass through that path length. For the direct method, the UCS of the sample was tested with a servo tester (WHY-200/10). It is worth noting that during the test, the samples were loaded with cracks of different widths in different directions, as shown in Figure 3 and Figure 4b.

### 3.2. Indirect Tensile Strength

An indirect tensile strength test was used to characterize the asphalt mixture in the tensile state [21], which can estimate the possibility of low temperature and fatigue cracking of asphalt concrete material under the action of thermal load and wheel load. Asphalt concrete samples underwent indirect tensile testing according to test method T0716-2011 of the technical specification [27]. A thin iron bar was placed parallel to the crack on the top surface of the sample, and a uniaxial compressive load was applied to the sample until the sample broke (Figure 4c). By applying a vertical load to the sample, this test produces a relatively uniform tensile along the plane where the crack is located, and fracture usually occurs in the same plane. The indirect (splitting) tensile strength is calculated as follows:

(2) $$S_{t}=\frac{2P}{\pi A}$$

where *P* is the load when the sample is damaged and *A* is the cross-sectional area of the sample parallel to *P*.

### 3.3. Static Creep Test

Creep stiffness modulus is an important parameter for evaluating the deformation performance of asphalt concrete. According to NCHRP Project 9-6(1) [28], the test procedure was carried out using a servo tester (WHY-200/10) under a fixed loading compressive stress (*σ_c_*) of 0.12 MPa (5–25% of the compressive strength without lateral limit), as shown in Figure 4d. The creep stiffness modulus is calculated using Equations (3) and (4):

(3) $$E_{cq(t)}=\frac{\sigma_{c}}{\varepsilon_{c(t)}}$$

*E_cq_*_(*t*)_ is the creep modulus at time *t*; *σ_c_* is the compressive stress (Pa) applied to the sample; and *ε*_(*c*(*t*))_ is the strain in the sample at time *t* (Calculation of creep stiffness modulus at 1, 10, 100, 500, 1000, 1500, 2000, 2500, 3000, 3500, and 3600 s).

(4) $$\varepsilon_{c(t)}=\frac{\Delta_{v}(t)}{\ell }$$

where ∆*_υ_*(*t*) is the uniaxial deformation of the sample in time *t* and ℓ is the average height of the sample.

### 3.4. SEM/EDS

The split samples were analyzed using a scanning electron microscope (SEM) and energy dispersive spectroscopy (EDS) to obtain the microscopic morphological characteristics and chemical composition of microbial mineralization products.

The electron microscope and energy dispersive spectrometer used in this experiment were both from Hunan Kewei Testing Technology Co., Ltd., and the parameters were as follows: the model of the electron microscope was JSM-7610FPlus with a magnification of 500~10,000 times; the model of energy dispersive spectrometer was ULTIM MAX 40, and the detection elements were mainly C, O, and Ca.

## 4. Analysis and Evaluation of the Effect of Repairing the Width of Small Cracks (≤1.5 mm)

### 4.1. Observation of Repair Effect

**Figure 5:**
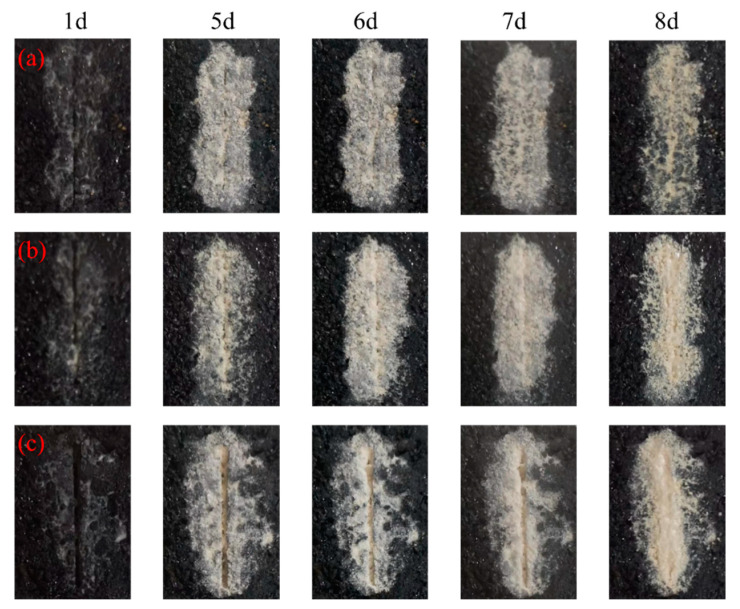
Filling situation of crack surface: (**a**) 0.5 mm; (**b**) 1.0 mm; and (**c**) 1.5 mm.

Figure 5 shows the top surface (cross section) of the asphalt concrete samples after one day and 5 to 8 days of MICP treatment. It can be observed that in the MICP-treated samples, the cracks gradually healed as the number of days of treatment increased. After five days of repair, the samples with crack widths of 0.5 mm and 1.0 mm (Figure 3a and Figure 5b) were basically healed, and visible cracks were still evident in the samples with crack widths of 1.5 mm (Figure 5c). After eight days of repair, the surface cracks of the samples were almost completely healed or sealed. In addition, the entire crack section of the sample was covered with precipitated calcium carbonate produced by the MICP process in the vicinity. The reason for the different distribution of white precipitates at the crack openings is that some organic matter produced during bacterial metabolism adheres the mineralization product particles together, thus forming dense, cohesive larger particles.

**Figure 6:**
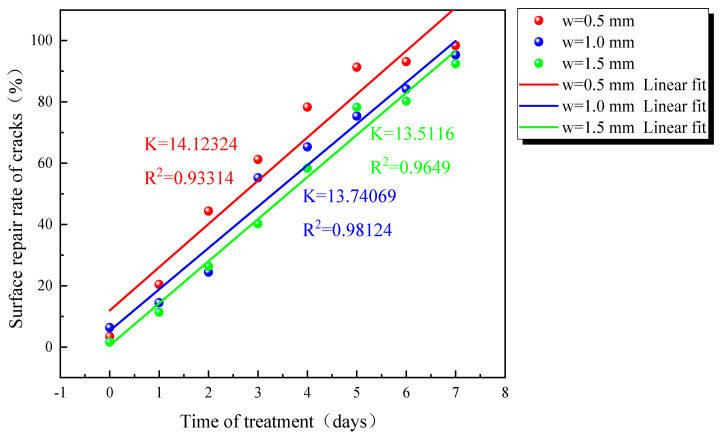
Percentage of fracture filling for different durations.

As Figure 6 shows the changing pattern of the surface repair rate and the fitted straight line in the repair process for different-width cracks. From the slope of the fitted straight line, it can be seen that the surface repair rate increases more rapidly for the sample with a crack width of 0.5 mm; the surface repair rate is the slowest for the sample with a crack width of 1.5 mm.

### 4.2. UPV

**Figure 7:**
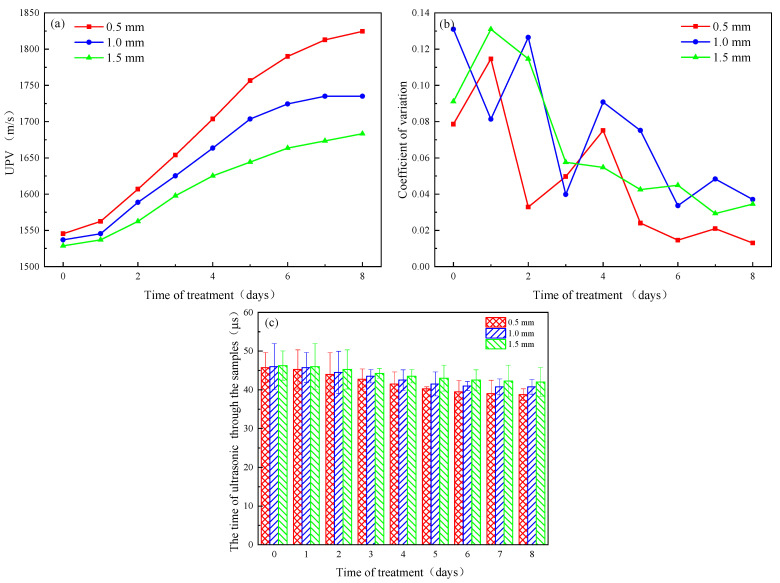
Ultrasonic velocity test results: (**a**) wave speed variation; (**b**) dispersion coefficient; and (**c**) sample ultrasonic treatment duration.

The greater the ultrasonic velocity, the better the repairing effect of asphalt concrete cracks. Therefore, the ultrasonic detection method can indirectly reflect the healing efficiency of asphalt concrete cracks. The results of the ultrasonic method for asphalt concrete before and after crack repair are shown in Figure 7a. After 8 d of healing, the acoustic wave velocity increased from 1545.36, 1536.96, and 1528.65 m/s to 1824.52, 1734.97, and 1683.33 m/s for initial crack widths of 0.5 mm, 1.0 mm, and 1.5 mm, respectively.

It can be seen that the ultrasonic velocity coefficient of variation for the three crack widths does not change much before and after the 8-day healing time (Figure 7b), and the changes are all within the range of 0.1. It is noteworthy that the coefficient of variation shows a decreasing pattern in general. The coefficient of variation for the cracks at a later stage of repair was lower than those for the cracks before repair, which may be due to the fact that the cracks were all better filled at the later stage of repair than at the earlier stage of repair, and thus the measured average wave velocities were more representative.

The asphalt concrete samples consisted of a solid and a gas phase because the asphalt concrete samples were sufficiently dry prior to the ultrasonic pulse velocity test. Due to the speed of propagation of the sound in solids being greater than that in air, when the cracks are filled, the solid phase of the asphalt concrete increases, the gas phase decreases, and the wave velocities show an increasing trend. The structural integrity of the 0.5 mm crack-width samples is greater than that of the 1.0 mm and 1.5 mm crack-width samples, and it is less difficult to form an effective ‘bridging’ effect on both sides of the crack than in the 1.0 mm and 1.5 mm crack-width samples, so the increase in wave velocity is most significant. The speed of the ultrasonic pulse increases as the crack width decreases, which is consistent with experimental expectations, and is in line with the results of Jongvivatsakul et al. [29] who showed the same pattern for the ultrasonic pulse velocities for the MICP repair of mortar cracks of different widths.

The above analysis shows that microorganisms can produce mineralization products for repairing asphalt concrete cracks of different widths, and the repairing effect is better for samples with smaller crack widths than those with larger crack widths.

### 4.3. UCS

**Figure 8:**
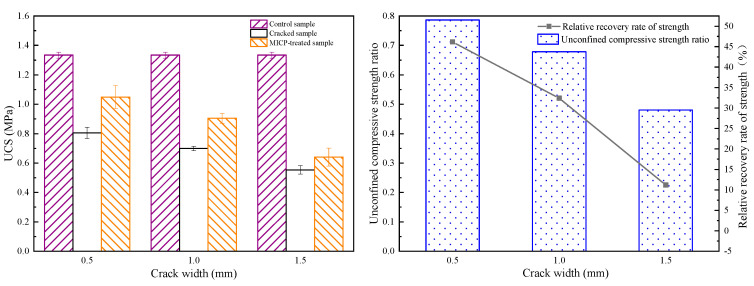
Compressive strength relationship for different crack widths under MICP treatment: (**left**) uniaxial compressive strength; and (**right**) strength ratio and recovery rate.

The compressive strength of the mortar samples is shown in Figure 8. The presence of cracks in the samples was the main reason why the compressive strength of asphalt concrete was lower than that of intact samples. The compressive strengths of cracked asphalt concrete samples were 0.80, 0.70, and 0.55 MPa when the crack widths were 0.5, 1.0, and 1.5, respectively, which were 39.84%, 47.37%, and 58.65% lower than those of the control. This is due to the high-stress concentration caused by cracks, which resulted in damage at lower stresses compared to the control samples. After treatment with bacterial healing agents, the available bearing area increased with the formation of CaCO_3_, thus allowing load transfer. The results showed that the stress concentration at the crack tip was reduced. As a result, the compressive strength of the cracked asphalt concrete increased to 1.05, 0.90, and 0.64 MPa. In addition, the compressive strength ratios were up to 78.94%, 67.67%, and 48.12% compared to the control samples. The increase in strength with crack healing is consistent with the increase in pulse velocity. Zheng et al. [30] and Qian et al. [31] confirmed that the higher the velocity of acoustic waves through the sample, the stronger the material.

The asphalt has a strong bond between the aggregate and the calcium carbonate, which helps to form a bridge between the two sides of the crack [32]. The UCS decreases with increasing crack width, which indicates that strength loss increases with cracking. In addition, smaller crack widths exhibit greater microbial utilization during MICP repair [33], such that samples with a crack width of 0.5 mm show a larger UCS after MICP repair.

### 4.4. ITS

**Figure 9:**
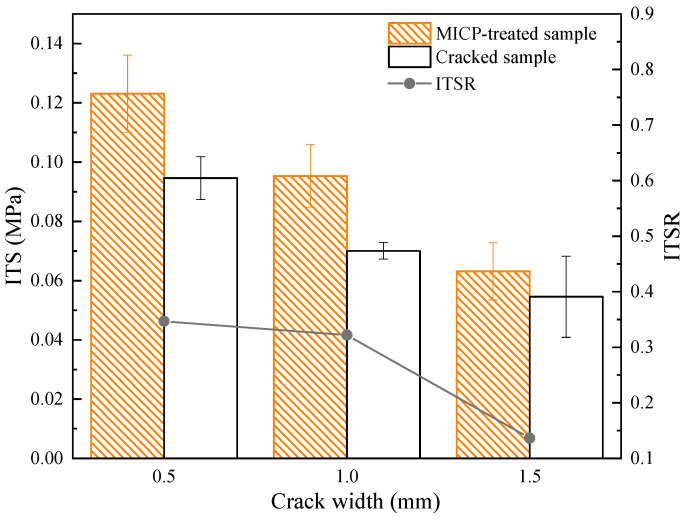
Indirect tensile strength relationship for different crack widths under MICP treatment.

The indirect tensile strength (ITS) of MICP-repaired cracks of different widths is shown in Figure 9. It can be seen that the ITS values of unrepaired samples decreased with the increase in crack width. The ITS values of the samples repaired by MICP increased from 0.091, 0.070, and 0.055 MPa to 0.122, 0.092, and 0.063 MPa, respectively, and the recovery of tensile strength reached about 34.68%, 32.19%, and 13.64%.

It is speculated that the lower value of ITS may be due to insufficient crack healing and the formation of an imperfect bond between cracked samples. The samples with larger crack widths repaired by MICP showed smaller ITS. One reason for this may be the lack of bonding between the cracks and the smaller size and microstructure of the CaCO_3_ distribution. The researchers found similar effects for potential applications in terms of indirect tensile testing following the bacterial repair of cracks in cement mortars [34]. Therefore, it can be concluded that the repair of asphalt concrete samples by MICP does increase the indirect tensile strength of the samples [35]. The mineralization products act as cementitious fillers in the cracks, and the ITS increase is more pronounced for small crack widths.

### 4.5. Permanent Deformation

**Figure 10:**
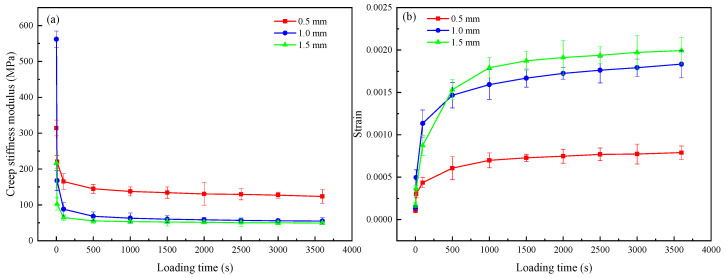
Static creep test: (**a**) creep stiffness modulus; and (**b**) strain-loading time curve.

The results of the static creep test are shown in Figure 10. It can be seen that the creep stiffness modulus has a large abrupt change within 100 s of loading, and after 500 s, the value of the creep stiffness modulus gradually stabilizes. The creep stiffness modulus decreases as the crack width increases. Notably, the creep stiffness modulus of asphalt concrete samples with crack widths w = 1.0 mm and 1.5 mm is slightly more prominent in the former than in the latter. In general, it is still consistent with the fact that the repaired creep stiffness modulus of the large crack width is smaller than that of the small crack width. This is because samples with large crack widths may have uneven filling as the cracks at the same depth are filled with mineralization products differently during microbial grouting.

The researcher, Manfro [36], found that the addition of calcium carbonate provides a resistance gain at the asphalt binder–aggregate interface, resulting in a modified asphalt mixture with greater resistance to permanent deformation. Samples with small crack widths and small voids between the two sides of the crack make it easier for MICP to achieve a relatively complete cementitious filling in the voids. They are easier to ‘bridge’, and the CaCO_3_ bridge formed is less likely to break in the middle, so it has a greater ability in preventing permanent deformation.

### 4.6. SEM/EDS Analysis

**Figure 11:**
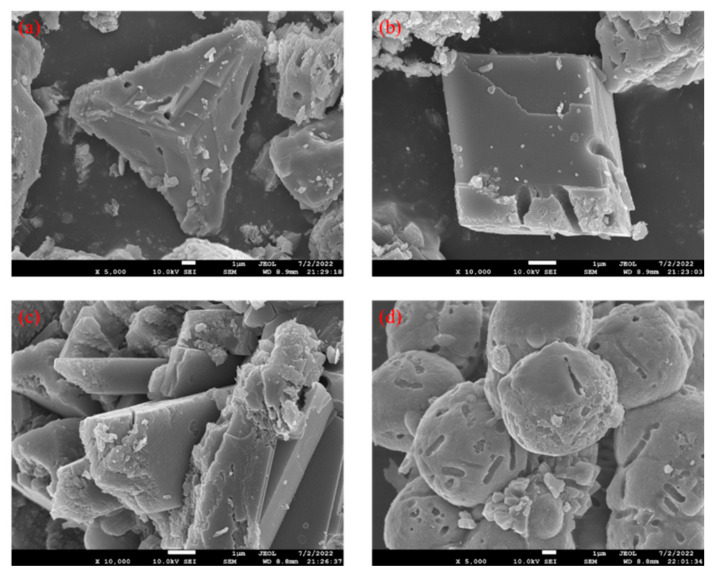
Scanning results of MICP product electron microscopy: (**a**) triangular cone; (**b**) parallel hexahedron; (**c**) prism; and (**d**) spherical-shaped.

SEM scans (Figure 11) show that the MICP mineralization products of this test have about four morphologies of crystallization. They are triangular conical, parallel hexahedral, prismatic, and spherical. Among them, the most common crystalline morphology is spherical.

**Figure 12:**
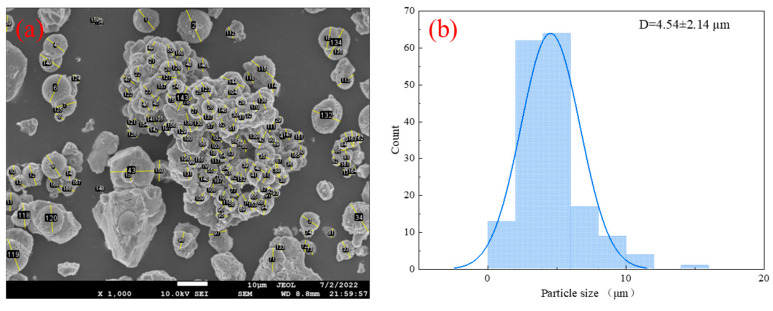
MICP product diameter measurement: (**a**) selected particles; and (**b**) particle size distribution.

The particle size distribution of the particles in Figure 12 was measured using Image J2 software, and the selected particles are the particle sizes observable on the image surface, as shown in Figure 12a. Figure 12b shows the distribution range of the particle size, from which it can be seen that the majority of the MICP products have a particle size distribution in the range of 0 to 10 μm, and the mean value of the diameter of the 170 particles selected was 4.54 μm.

**Figure 13:**
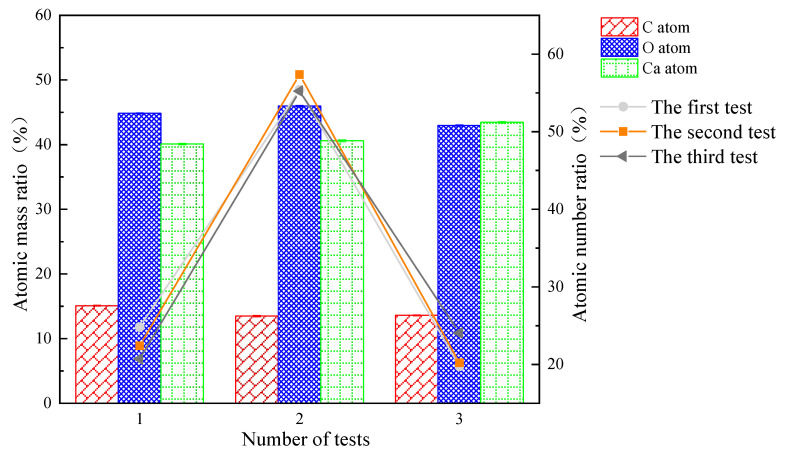
EDS scan results.

A total of three EDS scans were performed in this experiment, and the analytical results are shown in Table 3 and Figure 13. The results showed that the precipitates were mainly composed of C, O, and Ca, with an atomic number approximately close to 1:3:1 and an atomic mass ratio of approximately 1:3:3 (Figure 13). It can be inferred that the MICP production is calcite.

## 5. Analysis and Evaluation of the Effect of Repairing Large-Width Cracks (3 mm)

### 5.1. Effect of MICP Repair for Different Types of Fibers

**Figure 14:**
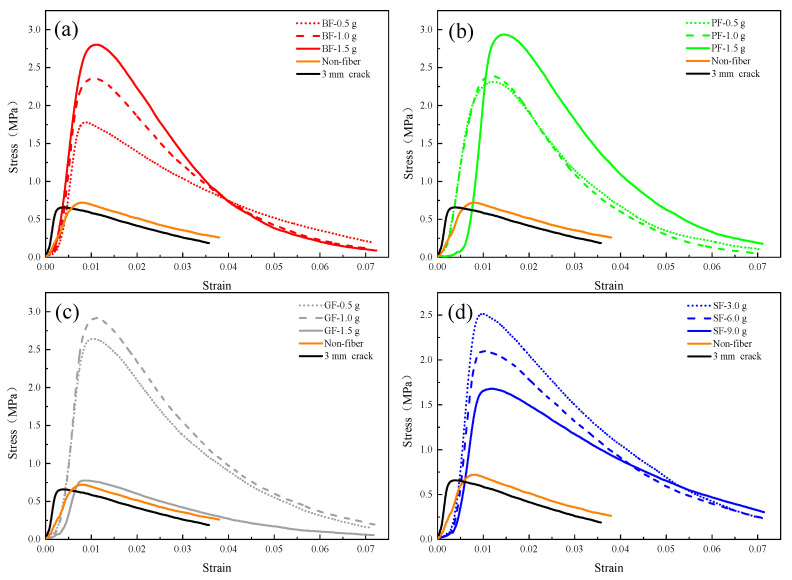
Stress–strain curves of repaired samples with different levels of fiber doping. (**a**) Basalt fibers (**b**) Polyester fibers (**c**) Glass fibers (**d**) Steel fibers.

Figure 14 shows the stress–strain curves of the samples with different fiber admixtures for repairing large-width asphalt concrete cracks. As a control, the stress–strain curves of the samples with 3 mm cracks without repair and without any fiber admixture are also shown in the figure.

Figure 14a illustrates that when the basalt fiber (BF) doping is increased from 0.5 g to 1.5 g, the strength increases, and the magnitude of the increase is close to about 0.45 MPa. In addition, the strain increases when the peak strength is reached. This indicates that the strength and toughness of the samples increased with the increase in basalt fiber doping during the MICP repair. Figure 14b demonstrates that when the doping of polyester fiber (PF) was increased from 0.5 g to 1.0 g, the strength increased, but not significantly, while the fiber doping was further increased from 1.0 g to 1.5 g, the strength increased relatively more significantly by about 0.54 MPa. This indicates that the small amount of basalt fiber (BF) and polyester fiber (PF) doping during MICP remediation is beneficial for bacteria to remain on them, and as a connecting bridge for MICP products, the fibers can form a bridging effect within the cracks for the integrity of the repaired samples.

Figure 14c shows that the strength of glass fiber (GF) increased by 0.27 MPa when increasing from 0.5 g to 1.0 g. In contrast to the pattern shown in Figure 14a,b, the strength did not increase but showed a significant decrease when increasing the fiber doping from 1.0 g to 1.5 g. The decreased strength was comparable to that of the MICP repair without any fiber doping. The reason for this phenomenon can be explained by the fact that the glass fiber admixture does help to improve the strength of asphalt concrete to some extent, the space inside the cracks of the samples is limited, and it is difficult for the fungus solution and the cementing solution to contact with it fully when the fiber admixture is too high, i.e., the MICP mineralization effect is negatively affected.

Figure 14d shows that during the MICP repair process, the strength gradually decreased as the steel fiber (SF) admixture increased from 3.0 g to 9.0 g, and the gradient of decrease was similar, about 0.4 MPa. This indicates that when the steel fiber admixture was too large, it squeezed too much space inside the cracks, and the effective bridging effect between the fiber and MICP products could not be formed.

**Figure 15:**
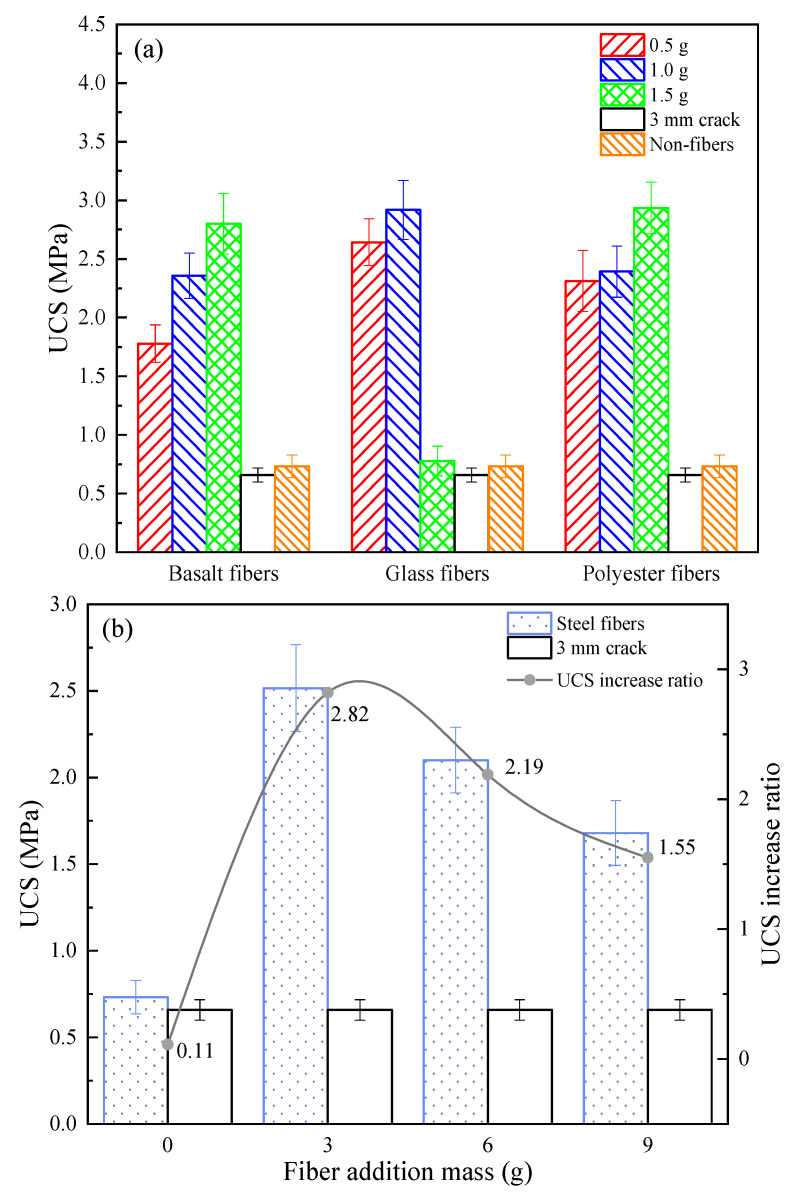
UCS with different fiber types and fiber doping: (**a**) BF, GF, and PF; and (**b**) SF.

Figure 15 visualizes the compressive strength of each sample with different fiber types and fiber mass for MICP repair. It can be seen that the measured compressive strengths differed from the previous sections when conducting the experimental study of repairing asphalt concrete samples with large-width cracks and were generally greater than those of the previous samples repairing cracks of different depths and small widths, which can be explained by the different placement of the samples when measuring the compressive strengths in this subsection compared to the previous subsections (shown in Figure 3). In addition, it is worth noting that the MICP repair without fiber doping showed an increase in strength compared to the samples with 3 mm cracks, but the increase was not significant, only 11.22%, indicating that when the crack width is greater than 3 mm, it is no longer appropriate to use a direct MICP repair. Scholars [37,38,39,40] have found similar effects when studying the coupling of MICP with each fiber.

The reasons for the above results can be inferred as follows:

(1) In the MICP repair process, the different fibers added in the cracks, even though they have the same quality, show different repair effects due to their different softness and volume, resulting in different distribution patterns in the cracks and different interaction relationships with the asphalt binder, aggregates, and MICP products.
(2) In the case of basalt fiber, its texture is hard, and it plays the role of strengthening the interconnection between calcium carbonate particles, and also provides the landing point for bacteria; in the case of polyester fiber, its texture is soft and it is more aggregated and distributed under the infiltration of slurry, which plays the local geotextile effect; for glass fiber, its softness and hardness are between basalt fiber and polyester fiber, and due to its longer length, it becomes entangled in the cracks. This winding effect can also strengthen the cementation between calcium carbonate particles, aggregates, and fibers to a certain extent; in the case of steel fibers, it has the hardest texture and the largest mass in the same volume, and the interlocking between fibers can also lead to the local siltation of calcium carbonate when repairing cracks via MICP due to the sinking through gravity, resulting in the effect with steel fibers where the MICP repair only occurs in a certain depth range of the cracks.

### 5.2. Investigation of Optimal Fiber Doping

**Figure 16:**
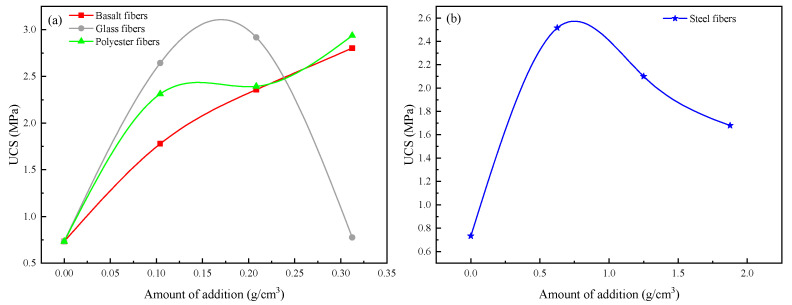
UCS–fiber doping fitting curve. (**a**) Basalt fibers, Glass fibers, Polyester fibers (**b**) Steel fibers.

Figure 16 shows the fitted curves of the doping mass of basalt fiber, glass fiber, polyester fiber, and steel fiber in the MICP process versus UCS, from which the fiber doping mass that has the best effect on UCS improvement can be deduced. The amount of addition for each fiber in this paper can be calculated using the following equation:

(5) $$D=\frac{m}{V_{c}}$$

where *m* is the mass of fiber added and *V_c_* is the crack volume.

It can be seen in the figure that the best doping mass of glass fiber is about 0.18 g/cm^3^, the best doping mass of steel fiber is about 0.83 g/cm^3^, and the best doping mass of polyester fiber and glass fiber is more than 0.31 g/cm^3^. The specific values are subject to further experimental study.

## 6. Discussion

The spherical calcium carbonate particles of MICP mineralization products have better fluidity and dispersion [41], which are easily attached near the crack surface and clustered with each other from particle to particle, allowing the cracks to be fully cemented and filled.

Since the propagation speed of acoustic waves in solids is greater than that in air, the denser the mineralization quantities in products, the greater the measured wave speed, and the better the filling effect of cracks [42]. After the cracks are filled by MICP products, the bridging effect is produced on both sides of the cracks [43]. The degree of bridging effect is different due to the different widths of the cracks. The direct manifestation of the bridging effect is the ultrasonic wave velocity, and the ultrasonic wave velocity of the sample with large crack width after MICP repair is smaller than that of the smaller crack width, indicating that the large crack width is not easy to form a significant bridging effect. The indirect manifestation of the bridging effect is UCS, and the study showed that adding fibers to the cracks could enhance this bridging effect [37]. Corresponding to the ultrasonic wave velocity test results, the UCS of the MICP-repaired samples decreased as the crack width increased.

The adhesion between the microbial mineralization products and the cracks of the asphalt concrete samples depended upon the compatibility between the roughness of the crack surface and the mineralization products, while the strength of the mineralization products depended on their own properties [44]. Since the crack surface roughness was almost the same for all samples, the tensile strength depended more on the direct adhesion between the mineralization products and the cracks for the samples with small-width cracks. The indirect tensile strength of the MICP-repaired samples increased when the crack width decreased, which can be explained by the stronger adhesion between the MICP mineralized products and the cracks for small-width cracks. In addition, the stronger adhesion also resists a greater resistance to permanent deformation under this adhesion in the static creep test.

In order to facilitate quantitative research, all of the repair work in this paper was carried out on regular cracks. However, in practice, regular cracks are almost nonexistent, and, therefore, the effect of irregular cracks on the repair effect needs to be considered. Irregular crack extensions make it difficult to control the percolation path of the biological slurry within the crack and make the repair more difficult and complex. In future research, the percolation channels of MICP slurry under complex fracture extension and the mechanism of MICP product attachment under such complex percolation can be further explored.

## 7. Conclusions

In this study, the effectiveness and mechanism of the (MICP) technique for repairing asphalt concrete with different crack widths were investigated through a series of experiments. Several conclusions were drawn as follows:

(1) The wave speed increased from 1545.36, 1536.96, and 1528.65 m/s to 1824.52, 1734.97, and 1683.33 m/s for crack widths of 0.5 mm, 1.0 mm, and 1.5 mm, respectively, and the uniaxial compressive strength for the MICP-repaired samples also increased compared to the unrepaired samples by 78.94%, 67.67%, and 48.12%, respectively. In addition, the recovery rates of indirect tensile strength were 34.68%, 32.19%, and 13.64%, respectively.
(2) The static creep test results showed that the creep modulus of the MICP samples was finally stabilized at 123.85 MPa, 54.83 MPa, and 49.83 MPa after 3600 s of constant pressure loading, indicating that the repaired MICP with a crack width of 0.5 mm had greater resistance to deformation.
(3) SEM and EDS images showed that the MICP product of this study was CaCO_3_, which was mainly in the form of spherical calcite. the particle size of CaCO_3_ was mostly concentrated within 10 μm, with an average value of 4.54 μm.
(4) The results of MICP combined with different fibers to repair large-width cracks showed that the addition of fibers contributed to the filling effect of the MICP product in the cracks of the as-built concrete. the UCS results showed that the optimum dose was about 0.18 g/cm^3^ for glass fibers, about 0.83 g/cm^3^ for steel fibers, and over 0.31 g/cm^3^ for polyester and basalt fibers.

The above findings indicate that the asphalt concrete samples with single cracks repaired with MICP showed some degree of recovery in performance. In general, the repair effect increased significantly as the crack width decreased. When the crack width was more extensive, the combined repair effect of MICP and fiber was better than the direct MICP repair.

## Figures and Tables

**Table 1:** Aggregate grading.

**particle size (mm)**	0–0.125	0.125–0.5	0.5–3	3–6	6–9
**Mass fraction (%)**	11.8	11.8	34.1	23.9	18.4

**Table 2:** Procedure for each group of tests.

Crack Widths		Whether to Add Fiber	Nondestructive Testing UPV	Destructive Testing		
				UCS	ITS	Creep
**Small widths**	**0.5 mm**	/	√	√	√	√
	**1.0 mm**	/	√	√	√	√
	**1.5 mm**	/	√	√	√	√
**Large width**	**3.0 mm**	√	/	√	/	/

Note: Three replicate trials were conducted for each group. ‘√’ means carried out, ‘/’ means not carried out.

**Table 3:** EDS test results.

Element	The First Test			The Second Test			The Third Test		
	Wt %	Wt % Sigma	Atomic %	Wt %	Wt % Sigma	Atomic %	Wt %	Wt % Sigma	Atomic %
**C**	15.09	0.09	24.83	13.47	0.10	22.41	13.59	0.15	20.71
**O**	44.83	0.12	55.40	45.95	0.14	57.37	42.97	0.17	55.23
**Ca**	40.08	0.10	19.77	40.58	0.12	20.22	43.44	0.12	24.06
**Total**	100	/	100	100	/	100	100	/	100

## Data Availability

The data that support the findings of this study are available upon request from the authors.

## Abbreviations

**Abbreviation**	**Definition**
MICP	microbially induced calcium precipitation
SEM	scanning electron microscope
EDS	energy dispersive spectrometer
CAEM	cold asphalt emulsion mixes
UPV	ultrasonic pulse velocity
UCS	uniaxial compressive strength
ITS	indirect tensile strength
BF	basalt fiber
PF	polyester fiber
GF	glass fiber
SF	steel fiber

## References

[1] Yang Z. (2018). Study on Multi-Scale Behavioral Characteristics of Asphaltbefore and after Aging. Ph.D. Thesis.

[2] Kim H., Wagoner M.P., Buttlar W.G. (2008). Simulation of Fracture Behavior in Asphalt Concrete Using a Heterogeneous Cohesive Zone Discrete Element Model. J. Mater. Civ. Eng..

[3] Ma Z., Liu L., Sun L. (2018). Investigation of top-down cracking performance of in-situ asphalt mixtures based on accelerated pavement testing and laboratory tests. Constr. Build. Mater..

[4] Cascione A.A., Williams R.C., Yu J. (2015). Performance testing of asphalt pavements with recycled asphalt shingles from multiple field trials. Constr. Build. Mater..

[5] Zhang Z., Sun J., Huang Z., Wang F., Jia M., Lv W., Ye J. (2021). A laboratory study of epoxy/polyurethane modified asphalt binders and mixtures suitable for flexible bridge deck pavement. Constr. Build. Mater..

[6] Shafabakhsh G., Ahmadi S. (2019). Reflective cracking reduction by a comparison between modifying asphalt overlay and sand asphalt interlayer: An experimental evaluation. Int. J. Pavement Eng..

[7] Moreno-Navarro F., Sol-Sánchez M., Rubio-Gámez M.C. (2015). The effect of polymer modified binders on the long-term performance of bituminous mixtures: The influence of temperature. Mater. Design.

[8] Fan T., Si C., Zhang Y., Zhu Y., Li S. (2023). Optimization Design of Asphalt Mixture Composite Reinforced with Calcium Sulfate Anhydrous Whisker and Polyester Fiber Based on Response Surface Methodology. Materials.

[9] Xue X., Gao J., Wang J., Chen Y. (2021). Evaluation of High-Temperature and Low-Temperature Performances of Lignin-Waste Engine Oil Modified Asphalt Binder and Its Mixture. Materials.

[10] Li H., Yu J., Wu S., Liu Q., Li Y., Wu Y., Xu H. (2019). Investigation of the Effect of Induction Heating on Asphalt Binder Aging in Steel Fibers Modified Asphalt Concrete. Materials.

[11] Khater A., Luo D., Abdelsalam M., Ma J., Ghazy M. (2021). Comparative Life Cycle Assessment of Asphalt Mixtures Using Composite Admixtures of Lignin and Glass Fibers. Materials.

[12] Wang W., Cheng Y., Tan G. (2018). Design Optimization of SBS-Modified Asphalt Mixture Reinforced with Eco-Friendly Basalt Fiber Based on Response Surface Methodology. Materials.

[13] Awuah F.K.A., Garcia-Hernández A. (2022). Machine-filling of cracks in asphalt concrete. Automat. Constr..

[14] Yin J., Pang Q., Wu H., Song W. (2018). Using a polymer-based sealant material to make crack repair of asphalt pavement. J. Test. Eval..

[15] Gnatenko R., Tsyrkunova K., Zhdanyuk V. (2016). Technological Sides of Crack Sealing in Asphalt Pavements. Transport. Res. Procedia.

[16] Wang Y., Kong L., Chen Q., Lau B., Wang Y. (2017). Research and application of a black rapid repair concrete for municipal pavement rehabilitation around manholes. Constr. Build. Mater..

[17] Sun X., Miao L., Wu L., Wang H. (2021). Theoretical quantification for cracks repair based on microbially induced carbonate precipitation (MICP) method. Cem. Concr. Comp..

[18] Cheng L., Kobayashi T., Shahin M.A. (2020). Microbially induced calcite precipitation for production of “bio-bricks” treated at partial saturation condition. Constr. Build. Mater..

[19] Peng S., Zhang K., Fan L., Kang J., Peng K., Wang F., Chen Z. (2020). Permeability Reduction and Electrochemical Impedance of Fractured Rock Grouted by Microbial-Induced Calcite Precipitation. Geofluids.

[20] Iqbal D.M., Wong L.S., Kong S.Y. (2021). Bio-Cementation in Construction Materials: A Review. Materials.

[21] Attaran Dovom H., Mohammadzadeh Moghaddam A., Karrabi M., Shahnavaz B., Attaran Dowom S. (2019). Investigation of the mechanical and physical properties of bio-modified cold asphalt emulsion mixtures by microbial carbonate precipitation. Int. J. Pavement Eng..

[22] Attaran Dovom H., Mohammadzadeh Moghaddam A., Karrabi M., Shahnavaz B. (2019). Improving the resistance to moisture damage of cold mix asphalt modified by eco-friendly Microbial Carbonate Precipitation (MCP). Constr. Build. Mater..

[23] Xin J., Pei J., Akiyama M., Li R., Zhang J., Shao L. (2019). A Study on the Design Method for the Material Composition of Small Particle-Size Asphalt Mixture for Controlling Cracks in Asphalt Pavement. Appl. Sci..

[24] Uhlmeyer J.S., Willoughby K., Pierce L.M. (2000). Top-Down Cracking in Washington State Asphalt Concrete Wearing Courses. Transport. Res. Rec..

[25] Brown E.R. (1999). Designing stone matrix asphalt mixtures for rut-resistant pavements (no. 425–430). Transportation Research Board.

[26] Bani Baker M.I., Abendeh R.M., Khasawneh M.A. (2022). Freeze and Thaw Effect on Asphalt Concrete Mixtures Modified with Natural Bentonite Clay. Coatings.

[27] Ministry of Transport of the People’ Republic of China (2011). Standard Test Methods of Bitumen and Bituminous Mixtures for Highway Engineering JTG E20–2011.

[28] Quintus H.V., Hughes C.S., Scherocman J.A. (1992). NCHRP asphalt-aggregate mixture analysis system. Transport. Res. Rec..

[29] Jongvivatsakul P., Janprasit K., Nuaklong P. (2019). Investigation of the crack healing performance in mortar using microbially induced calcium carbonate precipitation (MICP) method. Constr. Build. Mater..

[30] Wang Z., Ning J., Ren H. (2018). Frequency characteristics of the released stress wave by propagating cracks in brittle materials. Theor. Appl. Fract. Mech..

[31] Qian C., Zheng T., Zhang X., Su Y. (2021). Application of microbial self-healing concrete: Case study. Constr. Build. Mater..

[32] Pannem R., Chintalapudi K. (2019). Evaluation of Strength Properties and Crack Mitigation of Self-healing Concrete. Jordan J. Civ. Eng..

[33] Sun X.H., Miao L.C. (2020). Application of Bio-remediation with Bacillus megaterium for Crack Repair at Low Temperature. J. Adv. Concr. Technol..

[34] Kulkarni P.B., Nemade P.D., Wagh M.P. (2020). Healing of Generated Cracks in Cement Mortar Using MICP. Civ. Eng. J..

[35] Lu C., Ge H., Li Z., Zheng Y. (2022). Effect evaluation of microbial mineralization for repairing load-induced crack in concrete with a cyclic injection-immersion process. Case Stud. Constr. Mater..

[36] Manfro A.L., Melo J.V.S., Carpio J.A.V. (2022). Permanent deformation performance under moisture effect of an asphalt mixture modified by calcium carbonate nanoparti-cles. Constr. Build. Mater..

[37] Li H.F., Li Z., Liu Y. (2022). Effect of basalt fibers on the mechanical and self-healing properties of expanded perlite solid-loaded microbial mortars. J. Build. Eng..

[38] Yang D.F., Xu G.B., Duan Y. (2022). Self-healing cement composites based on bleaching earth immobilized bacteria. J. Clean Prod..

[39] Zhang D., Shahin M.A., Yang Y. (2022). Effect of microbially induced calcite precipitation treatment on the bonding properties of steel fiber in ultra-high performance concrete. J. Build. Eng..

[40] Zhao J.T., Tong H.W., Shan Y. (2021). Effects of Different Types of Fibers on the Physical and Mechanical Properties of MICP-Treated Calcareous Sand. Materials.

[41] Song C., Elsworth D., Zhi S., Wang C. (2019). The influence of particle morphology on microbially induced CaCO_3_ clogging in granular media. Mar. Georesour. Geotec..

[42] Zheng T., Su Y., Zhang X., Zhou H., Qian C. (2020). Effect and Mechanism of Encapsulation-Based Spores on Self-Healing Concrete at Different Curing Ages. ACS. Appl. Mater. Interfaces.

[43] Lin H., Suleiman M.T., Brown D.G. (2020). Investigation of pore-scale CaCO_3_ distributions and their effects on stiffness and permeability of sands treated by microbially induced carbonate precipitation (MICP). Soils. Found..

[44] Choi S.-G., Wang K., Wen Z., Chu J. (2017). Mortar crack repair using microbial induced calcite precipitation method. Cem. Concr. Comp..

